# 721 Defining Bleeding Characteristics in Frostbite Patients Managed with tPA

**DOI:** 10.1093/jbcr/irac012.275

**Published:** 2022-03-23

**Authors:** Jenna E Murphy, M Kenett Winters, Charlotte Rogers, Ellen Walter, Nichole Neumann, Lynn Weber, Alexandra M Lacey, Gopal Punjabi, Frederick W Endorf, Rachel M Nygaard

**Affiliations:** Hennepin Healthcare, Minneapolis, Minnesota; Hennepin Healthcare, Minneapolis, Minnesota; UTMB/Shriner’s Texas, Galveston, Texas; Hennepin County Medical Center, Minneapolis, Minnesota; Hennepin County Medical Center/Hennepin Healthcare, Minneapolis, Minnesota; Hennepin Healthcare, 715 Park Avenue, Minnesota; University of Wisconsin, Madison, Wisconsin; Hennepin Healthcare, Minneapolis, Minnesota; Hennepin Healthcare, Minneapolis, Minnesota; Hennepin County Medical Center, Minneapolis, Minnesota

## Abstract

**Introduction:**

Frostbite is caused by exposure to cold temperatures and can be a severe injury leading to hospital admissions, surgeries, or amputations. Disease progression involves endothelial injury, thrombosis, and tissue necrosis; therefore, management of patients involves a process of rewarming and restoration of blood flow to the affected area. Tissue plasminogen activator (tPA) is a thrombolytic agent that has demonstrated efficacy at restoring tissue perfusion in patients with frostbite. The goal of frostbite management with tPA is to salvage tissue without causing clinically significant bleeding, a documented adverse effect of tPA administration. The purpose of this study was to characterize specific bleeding complications associated with tPA administration. The secondary objective was to compare the rate of bleeding complications in frostbite patients treated with intravenous (IV) tPA to frostbite patients that did not receive tPA.

**Methods:**

This single center retrospective study included all adult patients with severe frostbite who presented between October 2013 and March 2020. tPA was given to patients per institutional protocol. To assess for bleeding events, patient charts were reviewed and any instance of bleeding was categorized based on severity. Bleeding was categorized as: 1) none, 2) mild: not clinically significant (bandage or moved IV site), 3) moderate: change of management (tPA stopped, enoxaparin held, or specialty consult), and 4) severe: included a change and intervention (transfusion, fasciotomy for compartment syndrome). Any change in management or any additional therapies used to control bleeding were documented, as well as the timing of bleeding in relation to tPA administration.

**Results:**

Over a 7-year period 209 patients were analyzed and 202 patients were included. For patients with bleeding events requiring intervention, the mean time to bleed was 105.5 hours (range 4 to 576 hours). Of these, 4 (3 transfusions and 1 fasciotomy for compartment syndrome) were temporally associated with tPA administration (within 24 hours). Two of the 4 patients had minor to moderate traumatic injury prior to admission, the 3rd patient had incomplete work-up at referring center that initiated tPA prior to transport, and the 4th patient was in restraints. Of the patients who did not receive tPA, 3.39% had a severe bleeding event requiring intervention compared to 6.99% of patients treated with tPA (P=0.516).

**Conclusions:**

Though there was a higher incidence of bleeding in tPA-treated patients, for the majority of patients studied, tPA was safe for the treatment of severe frostbite. Bleeding events occur in frostbite patients treated with or without tPA and warrant close follow-up for these infrequent complications.

